# A Review of the Functional Characteristics and Applications of *Aristotelia chilensis* (Maqui Berry), in the Food Industry

**DOI:** 10.3390/foods13060838

**Published:** 2024-03-09

**Authors:** Paula García-Milla, Rocío Peñalver, Gema Nieto

**Affiliations:** 1 Department of Food Technology, Nutrition and Food Science, Veterinary Faculty, Regional Campus of International Excellence “Campus Mare Nostrum”, University of Murcia, Campus de Espinardo, 30100 Espinardo, Murcia, Spain; ppaulagm@gmail.com (P.G.-M.); rocio.penalver@um.es (R.P.); 2 Carrera Nutrición y Dietética, Facultad de Ciencias de la Salud, Universidad Autónoma de Chile, Providencia 7500975, Chile

**Keywords:** Maqui, *Aristotelia chilensis*, functional food, antioxidants, polyphenols

## Abstract

The *Aristotelia chilensis* (Mol.) Stuntz, also known as Maqui, is an endemic berry native to southern Chile. It is a very popular berry for its nutritional attributes and health benefits, provided mainly by its polyphenols. This review aims to investigate the Maqui and its nutritional characteristics, its health benefits, and the application of Maqui in the food industry. This fruit provides 150 calories per 100 g of product and has a low protein content and a high fiber content. Its seeds contain monounsaturated fatty acids (MUFAs) and polyunsaturated fatty acids (PUFAs); however, its most outstanding feature is its high value of bioactive compounds, mainly anthocyanins, indole alkaloids and flavonoids, coumarins, caffeic and ferulic acids, and delphinidin 3-O-β-glucoside, the latter being the most representative, providing Maqui with high antioxidant activity. Maqui is considered a fruit of high interest as a nutraceutical product for the control and prevention of ongoing diseases, and among its benefits, we can highlight glycemic and metabolic control; the control and prevention of obesity, cancer, cognitive decline, and dementia; the prevention and treatment of bone structure alterations; prevention against oxidative stress, particularly in cigarette smoke-induced stress. In addition to its nutraceutical use, Maqui has been used in the food industry to improve the shelf life (by controlling lipid oxidation) and nutritional value of food products and as a substitute for synthetic additives. In addition, the inclusion of Maqui improves the organoleptic and sensory characteristics of foods. The incorporation of this fruit has been observed mainly in drinks, meat products, bakery products, and milk products. Evidence has shown that Maqui consumption, as well as products with Maqui added, have a good acceptability and exert benefits on people’s health. Knowledge about the application of Maqui in food will allow us to create new nutraceutical and food products that improve their nutritional and functional value.

## 1. Introduction

**Figure 1:**
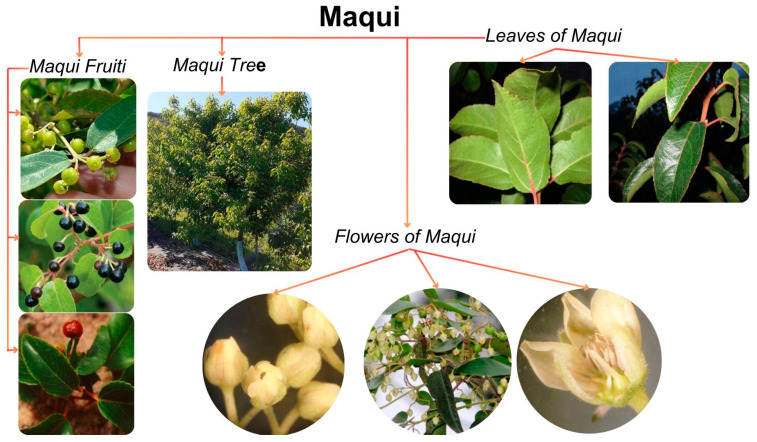
Maqui tree with its fruit, leaves, and flowers [4,13].

The *Aristotelia chilensis* (Mol.) Stuntz, also known as Maqui, is a plant native to Chile that is distributed in tropical and temperate Asia, Australia, the Pacific area, and South America [1,2]. It is an endemic tree present in Chile and in the adjacent regions of southern Argentina; it can be found in the north of Chile from the province of Limarí up to the province of Aysén in southern Chile. Maqui belongs to the *Elaeocarpaceae* family and is a plant that blooms from October to December, and it is harvested once a year, from December to February [3]. The Maqui is a thin tree whose girth measures from 30 to 35 cm and reaches a height of 10 m; it is a dioecious species; that is, it has male and female flowers of a pale-yellow color, with a diameter from 5 to 6 mm. Its fruit is an edible, round, dark purple berry whose diameter is 5 mm (Figure 1). Maqui berries contain a rich variety of anthocyanins, including delphinidins, powerful antioxidants found in abundance in the standardized Maqui berry extract [4].

According to research, Maqui has 19 polyphenolic compounds identified as anthocyanins (eight compounds), flavonols (10 compounds), and ellagic acid, with anthocyanins being the most predominant of the delphinidins, while quercetin derivatives were the most predominant flavonols [5]. These bioactive compounds have a strong antioxidant power, which is also associated with anticancer, antimicrobial, anti-inflammatory, and inhibitory activity on the enzymes that participate in metabolic syndrome [6,7], making Maqui a fruit of interest for health [4].

Despite the research associated with its benefits due to the contribution of its bioactive compounds, the bioavailability of anthocyanins has been questioned and is estimated at around 1% [8,9]. Possibly, this limitation in bioavailability may be due to factors such as cellular uptake in the intestine, a low absorption rate, and limited stability during the passage through the intestinal tract [10,11]. However, a study of 12 subjects observed a significant increase in plasma values of anthocyanins after ingestion of Maqui berry extract [4]. There is extensive discussion on the health advantages within the scientific community, which allows the use of this fruit as a medicinal alternative and a potential treatment for highly prevalent diseases, as well as in their prevention [12].

Given the interest presented by this native Chilean fruit, this review aims to investigate the functional characteristics and applications of *Aristotelia Chilensis*, including its applications in the food industry.

## 2. Nutritional Characteristics of Maqui Fruit

It is important to note that nutritional contributions will present variations depending on whether Maqui fruit is dried, fresh, or prepared as juice; it even shows differences according to the type of drying process, with freeze-dried Maqui being the most commonly sold [1,14].

According to Schmidt Hebbel [15], the nutritional composition of Maqui (Table 1) provides 150 calories per 100 g of edible portion. Its protein contribution is low, with 0.8 g/100 g; however, non-nitrogenous extractives, which correspond to carbohydrates and other constituents such as gel and fiber are quite significant. Maqui stands out for having the highest fiber content when compared to other berries like murtilla, blueberry, raspberry, and blackberry.

In a study that assessed the nutritional composition of the Maqui seed, it was found that it had a crude protein contribution of 9.24%, much higher than that reported by Schmidt [15] for the Maqui fruit. Maqui juice has a low protein and total carbohydrate content, according to a study published by Araneda et al. [16].

In addition, it has been reported that it contains calcium, phosphorus, iron, and potassium (Table 1) [13]. Furthermore, it has a lower ash content, as its juice is made with the Maqui extract and does not include the whole fruit [16].

The Maqui seeds showed a 2.06% ash content [17], similar to what was found in grape seeds, where Elagamey et al. [18] obtained ash values ranging from 2.52% to 2.68%.

Issis Quispe et al. [1] found that dried Maqui has total levels of fiber levels that vary between 53.3 and 64.5 g/100 g depending on whether it is fresh or dried. Of these values, insoluble fiber accounts for 50.8 to 58.2 g/100 g, representing up to 90% of total dietary fiber. According to Quispe-Fuentes et al. [1], this is due to the high ratio between pulp and seeds in Maqui berries.

An interesting study analyzed the ether extract of Maqui and mainly indicated that its seed has a high oil content [17]. In the research by Quispe-Fuentes et al. [1], 11 different types of fatty acids were identified, including six SFAs (saturated fatty acids), two MUFAs (monounsaturated fatty acids), and three PUFAs (polyunsaturated fatty acids), in both fresh and dried Maqui (Table 2). The main fatty acid was C18:2 (n-6), which represented 45.41% of the total, followed by C18:1 (n-9) (34.2%), C16:0 (9.49%), C18:0 (2.92%), and C18:3 (n-3) (2.12%) (Table 2) [16]. Different results were obtained in a study by Brauch JE et al. [14], in which lipids vary in most of the fatty acids found.

**Table 1:** Nutritional composition of Maqui fruit.

Nutrient	100 g of Edible Portion	Ref.
Energy (kcal)	150	[13,15]
Proteins (g)	0.8	[13,15]
Lipids (g)	2.9	[14]
SFAs %	13.6 ± 0.01	[16]
MUFAs %	35.25 ± 0.07	[16]
PUFAs %	47.78 ± 0.32	[16]
PUFAs/SFAs %	3.5	[16]
NNEs (g)	40.8	[13,15]
Total dietary fiber (g)	53.31	[16]
Insoluble dietary fiber (g)	50.85 ± 1.33	[16]
Soluble dietary fiber (g)	2.46 ± 0.60	[16]
α-Tocopherols (μg/g)	4.5 ± 0.1	[16]
Ash (g)	1.2	[13,15]
Calcium (mg)	87	[13,15]
Phosphorus (mg)	44	[13,15]
Iron (mg)	30.5	[13,15]
Potassium (mg)	296	[13,15]

NEs: non-nitrogenous extractives (by difference), SFAs: saturated fatty acids; MUFAs: monounsaturated fatty acids; PUFAs: polyunsaturated fatty acids.

**Table 2:** Composition of fatty acids in dried Maqui.

Fatty Acid g/100 g	Araneda et al. [16]	Brauch et al. [14]
Lauric acid C12:0	0.27 ± 0.01	0.49 ± 0.01
Myristic acid C14:0	0.66 ± 0.01	1.075 ± 0.02
Pentadecanoic Acid C15:0	n/i	0.02 ± 0.00
Palmitic acid C16:0	9.49 ± 0.04	8.70 ± 0.04
Stearic acid C18:0	2.92 ± 0.01	3.29 ± 0.05
Arachidic acid C20:0	0.21 ± 0.01	0.13 ± 0.00
Behenic acid C22:0	0.12 ± 0.08	0.19 ± 0.00
Lignoceric Acid C24:0	n/i	0.13 ± 0.01
Total SFAs	13.66 ± 0.08	n/i
Palmitoleic acid C16:1 (n-7)	0.33 ± 0.01	0.37 ± 0.02
Oleic acid C18:1 (n-9)	34.92 ± 0.06	33.28 ± 0.24
Total MUFAs	35.25 ± 0.07	n/i
Linoleic acid C18:2 (n-6)	45.41 ± 0.05	46.31 ± 0.09
Alpha-linolenic acid C18:3 (n-3)	2.12 ± 0.03	2.09 ± 0.03
Eicosapentaenoic acid C20:5 (n-3)	0.26 ± 0.24	n/i
Total PUFAs	47.78 ± 0.32	n/i

n/i: no information. SFAs: saturated fatty acids; MUFAs: monounsaturated fatty acids; PUFAs: polyunsaturated fatty acids.

## 3. Bioactive Compounds of Maqui Fruit

Polyphenols are antioxidant substances that are characterized by the presence of one or more phenolic rings and originate through biosynthesis as a product of the secondary metabolism of plants [19].

These can be classified in many ways due to their structural diversity; however, according to their chemical structure, they are classified into two large groups: flavonoids and non-flavonoids (Table 3) [19].

On the other hand, tannins are natural substances found in the plant world and are part of the polyphenol family [19]. They are highly hydroxylated molecules that can form insoluble complexes with carbohydrates and proteins and are responsible for the sensation of astringency perceived in foods.

Vegetable tannins can be subdivided into two main groups: hydrolysable tannins and condensed tannins (Table 3) [20], the latter being responsible for providing astringency to vegetable foods, when these tannins have a molecular weight between 500 and 3000 d.

Polyphenols play important functions in foods; they provide organoleptic characteristics such as color, bitterness, astringency, and aroma [19]; moreover, they have a high antioxidant activity, even higher than that of vitamins C and E [19].

A study employing a bioassay-guided approach to investigate the cytoprotective and antioxidant compounds in the Maqui berry resulted in the isolation and full identification of various phenolics. These included anthocyanins, flavonoids, acetophenone/benzaldehyde derivatives, simple phenolics, furfural derivatives, and a citric acid derivative [21]. Notably, work by Li et al. [21] (2017) marked the pioneering effort in isolating and individually testing Maqui berry constituents in a range of antioxidant assays. The study revealed a robust hydroxyl radical scavenging effect, and certain compounds demonstrated quinone reductase induction effects [21].

In a comparative study conducted by Speisky et al. [22], which determined the antioxidants of different fruits of the south Andes of South America, the Maqui berries exhibited the second-best antioxidant activity as determined by the ORAC assay in comparison with apples, cherries, and avocados. Additionally, Ruiz et al. [23] provided insights into the hydroxycinnamic acid (HCA) and flavonoid composition of Maqui fruit. The study also compared various extraction techniques for the quantification of these constituents (solid phase extraction using a mixed-mode cation-exchange cartridge and selective precipitation; and HPLC-DAD-ESI-MS/MS was applied to the extraction and identification of the main hydroxycinnamic acids and flavonols) [23].

Maqui fruit has been described as having high values of bioactive compounds [24], mainly anthocyanins, indole alkaloids and flavonoids, coumarins, caffeic and ferulic acids, and delphinidin 3-O-β-glucoside, the latter being the most representative [12].

According to research findings, Maqui has a high content of polyphenols in the different parts of the plant, both in the fruit and seeds, even when prepared as juice (Table 4) [19], which makes it very versatile for consumption and highly beneficial for health due to its high antioxidant capacity (Table 5), containing anthocyanins, phenols, flavonoids, among other bioactive compounds [25,26].

**Table 3:** General classification of tannins and polyphenols.

Polyphenols Classification [19]		Tannins Classification [27]	
Flavonoids (C_6_-C_3_-C_6_)	Non-Flavonoids	Proanthocyanidins or Condensed Tannins	Hydrolyzable or Pyrogallic Tannins
Formed by 2 benzene groups linked by a tricarbonate bridge. Subgroups: Anthocyanins. Flavones, flavonones, flavanols and flavanonols. Flavanols, condensed tannins, and lignans.	There are two subgroups among them: Non-carboxylic phenols: C_6_, C_6_-C_1_, C_6_-C_3_. Phenolic acids: C6-C_1_ benzoic acid and C_6_-C_3_ cinnamic acid derivatives.	Also initially called leucoanthocyanins, they exist as soluble oligomers, with 2 to 6 phenolic flavan-3-ol cores (catechin, epicatechin, epigallocatechin, or epigallocatechin 3-O-gallate), or as insoluble polymers.	They are esters of phenolic acids (gallic and ellagic acids) with a sugar (usually glucose) or a polyol.

**Table 4:** Polyphenol content in the different presentation forms of Maqui.

	Polyphenols (mg GAE/g)	Reference
Maqui Seeds	1670	[17]
Maqui juice	993.21 ± 54.87	[16]
Freeze-dried Maqui	49.74 ± 0.57	[13]
	53.3 (5.8 d)	[25]
Maqui leaves	69.0 ±0.9	[28]
Maqui fruit	45.7 ± 1.1 1664	[28]
Maqui stems	25.8 ± 0.3	[28]
Aqueous extract of Maqui	65.53	[29]

mg GAE: milligrams of gallic acid equivalent per gram.

**Table 5:** The amount of anthocyanins, flavonoids, and antioxidant capacity of the Maqui fruit according to the DPPH, ORAC, and FRAP methods.

Method	Antioxidant Capacity	Reference
DPPH (mg TE/g)	2.81 ± 0.04	[13] *
FRAP (mg TE/g)	25.2 ± 0.38	[13] *
ORAC (mmol TE/g)	0.3 ± 0.1	[25] **
Anthocyanins and Flavonoids		
Anthocyanins		
Delphinidin3-O-sambubioside-5-O-glucoside	4.01 ± 0.01	[30] *
Delphinidin3.5-O-diglucoside	3.51 ± 0.02	[30] *
Cyanidin3.5-O-diglucoside + Cyanidin 3-O-sambubioside-5-O-glucoside	1.76 ± 0.01	[30] *
Delphinidin 3-O-sambubioside	1.90 ± 0.02	[30] *
Delphinidin 3-O-glucoside	4.29 ± 0.04	[30] *
Cyanidin 3-O-sambubioside	0.05 ± 0.00	[30] *
Cyanidin 3-O-glucoside	1.07 ± 0.00	[30] *
Total Anthocyanins	16.59 ± 0.04	[30] *
Flavonoids	68.04 ± 24.63	[26] *

mg TE: milligrams of Trolox equivalents per gram. mmol TE: millimoles of Trolox equivalents per gram. * Freeze-dried Maqui. ** Maqui fruit.

## 4. Health Benefits of Maqui

The antioxidant capacity of Maqui has been of great interest within the scientific community due to its beneficial effects on health [31], the most prominent of which are improvements in memory and cognition, the contraceptive effect, the possible favorable effect on inflammatory bowel disease, liver lipogenesis, among others shown in Table 6.

Cognitive decline is a geriatric syndrome of major interest since it affects the quality of life of the elderly. Cognitive domains may be affected by different factors [32,33], with diet being one of them. The brain needs nutrients to function, such as amino acids, vitamins, fats, and carbohydrates [34], since they influence brain metabolism. However, this is not all, as antioxidants are very important for brain health.

According to a study on rats, which aimed to evaluate the protective effect of a Maqui extract (ME) in brain regions associated with cognition, it was determined that the administration of 50 and 100 mg/kg of the extract was effective in the prevention of cognitive deficit in rats exposed to 0.25 ppm of ozone. Researchers found that prolonged exposure to ozone can produce a state of oxidative stress, which in turn results in alterations in brain dynamics and affects memory and learning; however, this brain condition could be countered by the consumption of the Maqui berry due to its high antioxidant activity, which demonstrated a decrease in oxidative stress markers [35].

In addition, a double-blind randomized clinical trial showed similar results when participants were administered a standardized ME (162 mg anthocyanins) or a matched placebo, three times a day for a period of 4 weeks. Results showed that the supplementation with the extract was associated with a reduction in the levels of oxidized low-density lipoprotein and urinary F2-isoprostanes, suggesting that its use may improve oxidative status in healthy adults, as well as in overweight individuals and smokers [36]. Similar results were obtained in a 3-month trial including 31 subjects who were treated with a delphinidin-rich Maqui berry extract. Subjects showed a significant reduction in LDL after the treatment (*p* = 0.001) and a reduction in VLDL values after one month; however, the latter experienced a subsequent rise, showing no statistical difference. On the other hand, an important effect observed in this study was that glycosylated hemoglobin decreased from 5.65 ± 0.09% (SE) to 5.50 ± 0.08% (*p* = 0.084) after one month [37]. A similar experiment used the same delphinidin-rich Maqui extract and concluded that this extract may be an excellent ally in the glycemic control of diabetic individuals and insulin-resistant subjects [37].

The Maqui extract (ME) might be an excellent treatment for preventing cardiovascular diseases [38], since besides having an impact on lipid and glycemic control, it might reduce platelet aggregation according to a study in which researchers evaluated blood plasma using unripe fruits [39]. Another beneficial effect on cardiovascular and brain health might be related to the prevention and/or treatment of depression. In a study conducted on a 5-week-old male mouse, an ME was administered, and then the subjects behavior was evaluated after an ischemic stroke; the results showed a significant decrease (*p* < 0.05) in the intake of sucrose solution and a significant increase (*p* < 0.05) in water intake when compared to the controlled group; however, the extract mitigated the decrease in pleasure in mice, since it showed a dose-dependent increase in sucrose and a dose-dependent decrease in water in the intervened group. Despite this fact, the team concluded that Maqui, because of its antioxidant activity, could improve mouse behavior as it showed an effect similar to that of pharmacological treatment [40].

Tobacco use is one of the environmental or external factors that may increase oxidative stress, causing high-prevalent diseases. According to evidence, cigarette smoke is associated with increased oxidative stress, which in turn increases altered osteoblast differentiation and inhibits the mineralization process [41]. A group of researchers conducted an in vitro study that evaluated the antioxidant effect of a commercial delphinidin-rich ME on primary human osteoblasts obtained from the department of traumatology of a local medical center. The tissues were harvested and exposed to cigarette smoke to subsequently receive treatment with ME with a minimum of 25% delphinidins. Results showed that high concentrations of ME above 25 μg/mL have a toxic effect on human osteoblasts, while physiological concentrations indicated at 1.56 μg/mL have no negative effects on cells. Moreover, these physiological concentrations can reduce oxidative stress caused by cigarette smoke, having a preventive effect; in conclusion, physiological doses can effectively protect osteoblasts [41]. Such results are in line with other studies that found an improvement in the function of osteoblasts exposed to cigarette smoke, in the presence of ME concentrations similar (1.5 μg/mL) to those described in the previous study [42].

The effect of Maqui on bone structure has also been evaluated in in vivo studies on mouse (C57BL/6J) models with factor-kappaB Ligand (sRANKL)-induced and ovariectomy-induced osteopenia. Mice were tube-administered ME for 7 days prior to the administration of sRANKL and then for 14 days after the treatment. Results showed that ME stimulated osteoblast cell differentiation in an in vitro culture and stimulated the proliferation of MC3T3-E1 cells, significantly increasing mineral deposition on day 16 of the treatment; in addition, microcomputed tomography and analyses of femurs demonstrated that ME significantly increased the ratio of bone volume to tissue volume, concluding that it can be used as an agent for the prevention of bone loss [43].

Both oxidative stress and tobacco-induced inflammation can be controlled by the intake of Maqui, as confirmed by another study on 15 asymptomatic smokers with mild cigarette intake, who were given 2 g of extract twice a day for a period of two weeks. Results showed that at baseline, Hydrogen peroxide (H_2_O_2_, reactive oxygen species) concentrations were higher and IL-6 concentrations were lower in smokers than in non-smokers; after the intervention, H_2_O_2_ levels significantly decreased (*p* < 0.002) and IL-6 increased (*p* < 0.004), showing that the use of Maqui can normalize IL-6 and H_2_O_2_ concentrations in individuals with smoking habits [44].

Another study found that IL-6 levels, as well as tumor necrosis factor-α levels, were down-regulated and IL-4 levels were up-regulated when conducting in vivo experiments using ME on mice exposed to UVB radiation, suggesting that Maqui is additionally an effective agent against UVB-induced photodamage [45].

The benefits of Maqui on intestinal health were examined in an in vivo study on an inflammatory bowel disease model (ulcerative colitis) induced by dextran sodium sulfate. Researchers used 6-week-old mice and found a decrease in inflammatory bowel disease indexes in blood serums (*p* < 0.005) after 8 weeks of intragastric treatment with Maqui extract at doses of 50, 100, and 200 mg/kg. Furthermore, intestinal histopathological damage was significantly alleviated, and the expression of occluding was increased (*p* < 0.05); in addition, ME improved the gut microbiota of treated mice [32]. These results are similar to the study by Palta et al. [33], who also used an ME to evaluate its potential effect on inflammatory bowel disease, concluding that it is a nutraceutical agent with physiological benefits for the treatment of this disease.

These findings, as well as the available literature reviews, support the therapeutic and preventive activity of the Maqui berry in various diseases or clinical conditions that may affect people, making it an excellent nutraceutical that may be used in most individuals and in different clinical contexts.

**Table 6:** Evidence on the biological effects of Maqui.

Physiological Role	Design	Results	Conclusion	Reference
Cognitive performance	An in vivo study was conducted using male Sprague Dawley rats weighing between 250 and 300 g, exposed to O_3_ at a constant dose of 0.25 ppm for 4 h per day. The rats were randomly divided into seven experimental groups, with the administration of 50 and 100 mg/kg of the aqueous extract of Maqui berry.	Administering 50 and 100 mg/kg of the aqueous extract of Maqui berry proved effective in preventing cognitive deficits induced by chronic exposure to ozone.	A correlation exists between cognitive protection and a reduction in oxidative stress markers.	[35]
Antinociceptive effects	In an in vivo study with Swiss Webster male mice (25–30 g), the intervention group was enterally and parenterally administered Maqui diluted in water, at a volume of 0.1 mL per 10 g of body weight.	All doses exhibited significant inhibition of nociception at the central stage, achieving an effect comparable to the reference drug. This inhibition was also observed during the inflammatory stage, where Maqui, at doses of 62.5, 125, and 250 mg/kg, completely inhibited nociceptive behavior throughout the evaluated time, surpassing the response induced by the reference drug.	A modest consumption of this berry could serve as a potential natural alternative or complement to the use of conventional drugs for analgesic therapy.	[30]
Therapeutic impact on inflammatory bowel disease	An in vivo study with C57BL/6 male mice that were administered Maqui berry water extract in concentrations of 50, 100, and 200 mg/kg and compared to a controlled group. To induce the disease, researchers used dextran sodium sulfate (DSS).	Maqui extract considerably reduced the expression of COX2 and IL-6 in LPS-stimulated RAW 264.7 cells. Moreover, the inflammatory bowel disease index decreased in colon tissues in the treatment group compared to the model group (*p* < 0.05), and Maqui extract alleviated the imbalance of gut microbiota caused by DSS injury.	Maqui contributes to a therapeutic role in ulcerative colitis by exerting anti-inflammatory effects, alleviating immune stress, and regulating gut microbiota.	[46]
Antioxidant effect on human osteoblasts affected by cigarette smoke	An in vitro study on human bone cells collected from the Department of Traumatology, BG Unfallklinik Tübingen. Samples were exposed to cigarette smoke and delphinidins obtained from Maqui fruit of a Chilean producer; the extract was dissolved in the cell culture medium.	Results showed that high concentrations of Maqui extract are toxic for human osteoblasts, while physiological concentrations can reduce oxidative stress caused by cigarette smoke.	Maqui extract at physiological concentrations can effectively protect osteoblasts from oxidative stress-induced damage by activating the cells’ antioxidative defense system.	[46]
Effect on the reduction of cigarette smoke-induced cellular injury in a 3D bone coculture model	A three-dimensional co-culture system of human bone marrow mesenchymal stem cells was used; cells exposed to cigarette smoke that was prepared on the day of the medium change (concentration of 5%) were treated with 2 natural extracts (1.5μg/mL of Maqui, 50 μg/mL of ginseng, and 3.5 mM NAC were used as a positive control).	Co-cultures exposed to 5% cigarette smoke extract showed a significant reduction in mitochondrial activity (*p ˂* 0.0001) and total DNA (*p ˂* 0.0001), while NAC caused a significant increase in mitochondrial activity. The extracts showed a tendency to downregulate the expression of osteoclastic markers genes. There was a dramatic reduction in the ratio of sRANKL and OPG.	The extracts are considered a promising alternative for the bone health of orthopedic patients who smoke.	[42]
Bioactive phenolic components and their antiplatelet action	An intervention study where the antiplatelet activity of Maqui extract was determined using blood plasma obtained from 3 donors.	The extracts hinder platelet granule secretion by reducing the exposure of P-selectin and CD63 on the platelet membrane. Additionally, the formation of reactive oxygen species in platelets is diminished in the presence of Maqui extracts.	The extracts from leaves and unripe fruits exhibited a higher content of phenolic compounds, and this correlated with the observed antiplatelet potential in these extracts.	[39]
Cardioprotective effect	Maqui extract was obtained from its seeds and pulp, which were analyzed to examine their phenolic compounds. The antioxidant activity of phenolic compounds was evaluated. Rats weighing 250–300 g were used, and all the experiments were conducted at the same time, with rats being divided into a controlled group and intervention group.	The resulting cardiac injury was significantly reduced by the acute administration of one of the Maqui extracts, significantly reducing the increase in TBARS levels.	The reduction in TBARS concentration can be attributed to the high polyphenol content of the extracts.	[38]
Effects on post-stroke depression	An in vivo study with 5-week-old male mice weighing 20–25 g, which were provided food and water ad libitum. Mice were acclimatized 24 hrs. before behavioral tests, which were conducted at the same time each day. Mice were divided into 5 groups of 10 mice each, and a previously characterized Maqui extract was used for the intervention.	Results showed that the activity of Maqui extract is similar to that of conventional antidepressants, restoring normal behavior in intervened mice.	Maqui might be useful for supporting pharmacological therapy of depression, and its effect could be mediated by the modulation of stress.	[40]
Effect on glucose metabolic control	An intervention study in which 31 subjects were treated with a Maqui extract in a 3-month trial.	Glycosylated hemoglobin decreased from 5.65 ± 0.09% (SE) to 5.50 ± 0.08% (SE) (*p* = 0.084) after one month and 5.39 ± 0.08% (SE) (*p* = 0.010) after two months, while blood LDL levels showed a significant reduction in LDL after three months (*p* = 0.001).	It is noted that the use of a delphinidin-rich Maqui berry extract has potential health benefits, particularly regarding the improvement in glucose metabolism and lipid control	[37]
Effect on H_2_O_2_ and IL-6 concentrations among asymptomatic smokers	An intervention study in which 15 smokers with mild cigarette intake were administered 2 g of Maqui extract twice a day for two weeks. Results were compared with those of 8 healthy non-smoking subjects.	The Maqui extract led to a significant decrease in H_2_O_2_ (*p* < 0.0002) and an increase in IL-6 (*p* < 0.004) among smokers.	Maqui extract normalizes IL-6 and H_2_O_2_ concentrations in subjects with mild smoking habits.	[44]
Effect on bone loss	An in vivo study in an osteopenic mouse model in which mice were tube-administered a Maqui extract. A pre-treatment was applied 7 days prior to the induction of the disease, and then the treatment with the extract continued for 14 days.	The extract hindered osteoclastogenesis in primary bone marrow macrophages and inhibited pit formation by matured osteoclasts on dentine slices. Additionally, it significantly increased the ratio of bone volume to tissue volume.	The extract shows promise as a natural agent for preventing bone loss in osteopenic conditions, as it not only inhibits bone resorption but also stimulates bone formation.	[43]

## 5. Use of the Maqui in the Mapuche Population (Native People of Chile)

For centuries, the Mapuches (native people of Chile) have utilized these substances both as sustenance and for their medicinal and nutritional advantages, with historical records dating back to the time of their usage. In traditional medicine, an infusion of dried leaves is applied for wound healing, while a similar preparation from fresh leaves is employed to alleviate conditions like fever, diarrhea, and dysentery. Additionally, it serves to soothe inflammation of the pharynx and tonsils and alleviate mouth ulcers. The fresh leaf juice can be consumed or applied topically for these purposes.

Based on a historical analysis of Maqui berry’s composition, it has been determined that 100 g of the powder typically contains a total of 5 g of polyphenols. Taking this information into account, the recommended intake for a single portion is suggested to be 1.5–2 g (equivalent to 1/2 teaspoon) of Maqui berry powder. The maximum recommended intakes of Maqui berry powder as raw materials are detailed in Table 7 [47].

## 6. Applications of Maqui Fruit in the Food Industry

As previously mentioned, the health benefits associated with Maqui berry are often attributed to its abundance of bioactive compounds, particularly anthocyanins [48]. The interest in anthocyanins has risen significantly due to their association with a decreased risk of various chronic diseases. These compounds play a crucial role in promoting health by exhibiting antioxidant, anti-inflammatory, anti-cancer, and protective effects against metabolic, degenerative, and cardiovascular diseases [48]. Nonetheless, anthocyanins are inherently unstable and vulnerable to factors such as temperature, light, and pH conditions. Consequently, their bioavailability is recognized to be low, rendering their study complex [4]. To overcome this challenge, the intake of products rich in anthocyanins, incorporated into diverse food matrices, could provide protection against degradation caused by pH variations in different digestive stages. The structure and composition of the food matrix containing anthocyanins play a crucial role in either enhancing or hindering the release and stability of these compounds during digestion, thereby influencing their effectiveness. The bioactive compounds responsible for the claimed health benefits of Maqui berries must withstand the digestive process. In this sense, McDougall et al. [48] reported that when anthocyanin-rich raspberries are consumed alongside foods like bread, cereals, ice cream, or cooked meat, the anthocyanin content remains unaffected after gastric digestion.

Currently, the food industry has incorporated vegetable products into food composition, with the aim of providing a comprehensive product with benefits for consumers. In the case of Maqui fruit, it is mainly used in juices and in powdered, freeze-dried, and frozen forms, which are currently marketed both nationally and internationally. Various investigations have demonstrated that Maqui berry fruit was predominantly consumed in processed forms, such as dehydrated, jam, or juice. This preference can be attributed to distinct factors, including the fruit’s limited harvesting window and its notably short shelf life. According to the report *“Perspectivas del mercado internacional para el desarrollo de la industria del Maqui: Un análisis de las empresas en Chile” [International market perspectives for the development of the Maqui industry: An analysis of Chilean companies]* [49], there are 21 exporting and marketing companies of Maqui, of which 4 are exclusively engaged in the manufacture of beverages, with these being the second most traded product in terms of dollars, followed by Maqui powder.

Maqui juice can be marketed as natural juice, that is, raw and pure fruit juice, but also mixed with or added to other natural components, as is the case of lemon juice enriched with Maqui berries [50]. A study that analyzed blends of lemon juice mixed with different berries found that the blend including Maqui was the most interesting in terms of antioxidant capacity, showing reduced effects on acetylcholinesterase and butyrylcholinesterase; moreover, lemon juice mixed with Maqui can be useful to equilibrate redox balance in acute and intense exercise, it reduces glycemia levels in subjects of both sexes, and its composition has a lower glycemic index, with its properties remaining stable during preservation [50,51,52]. It is important to note the agent used to sweeten beverages or citrus-Maqui juices, since according to a study the use of non-caloric sweeteners could alter blood homocysteine levels when sucralose is used (*p* = 0.001); however, researchers observed a significant increase in IL-10 concentrations when the beverage was sweetened with Stevia [53]. Other research that analyzed the effect of a citrus-Maqui beverage with sweeteners on male and female consumers found that the use of Stevia regulated trans-ferulic acid levels in women, while sucrose regulated vanillic acid levels in men, concluding that sweeteners may influence the regulation of polyphenols in subjects who consume these drinks [54]. Along the same lines, a group of researchers designed an aniseed liquor-based beverage, which was originally produced in Navarra, incorporating Maqui berries. Results showed optimal organoleptic and sensory characteristics, with an attractive aroma and highlighting color; in addition, it exhibited a higher anthocyanin content during maceration and a higher antioxidant capacity [55].

Given the findings of earlier research indicating the potential utilization of bioactive compounds derived from herbs and plants in the development of innovative functional food products, Maqui emerges as a promising candidate for applications in functional foods and various industrial food sectors. Notably, Maqui offers health-promoting nutrients, positioning it as a crucial source of food security in economically challenged regions. This review aims to consolidate recent insights into the bioactive compounds derived from Maqui and explores their potential incorporation into food product formulations (Table 8).

In this sense, Maqui has also been used to improve the quality of beef patties. In a study where researchers compared control patties without antioxidants, patties added with synthetic antioxidants, and patties added with Maqui leaf powders in concentrations of 500, 1000, and 2000 ppm, it was found that the pH of the control beef patties increased during storage, while the pH of beef patties with synthetic and natural antioxidants decreased; furthermore, decreased lipid oxidation was observed in patties added with Maqui (at all doses) in comparison with the synthetic antioxidant. With respect to organoleptic characteristics, redness (a*) was affected at doses of 1000 ppm and 2000 ppm of Maqui powder; despite this, the incorporation of this product did not affect the general acceptability of the patties, concluding that this natural additive can be used in the formulation of beef patties [56,57].

Given the attractive color of the Maqui fruit, it has been tested as a natural colorant in yogurts. Researchers incorporated Maqui and murra (blackberry) to assess consumers’ perceptions; it was found that Maqui samples had the highest acceptability means, with 74% of consumers reporting that they “like it” and “like it very much” when concentrations of 8% were used, while a 64% rejection was reported when a Maqui concentration of 12% was used. Purchase decision was mainly observed in relation to the products including Maqui when compared to products added with murra (blackberry), concluding that concentrations of 4% and 8% Maqui could be used as natural colorants [58].

Maqui fruit can be employed in a variety of ways; according to a publication, it can also be an excellent additive to enhance the functional and nutritional properties of a product, as well as to improve organoleptic and sensory characteristics, and has been tried as an enhancer for thermal stability of avocado oil. Researchers used Maqui leaf extracts and concluded that methanolic extract has a better protective effect on thermal oxidation when the oil is heated at 120 °C for 336 h in an oven. Natural extracts, which are by-products (leaves), can be an alternative to stabilize oils subjected to high temperatures [59].

**Table 8:** Maqui’s application effect on product quality for bakery, meat, and milk products along with oils and juice.

Food	Parts Used	Maqui Application/Concentration Used	Main Results/Conclusions	Reference
Cookies	*Maqui* By-products (seeds and skin)	Substituting wheat flour in cookie formulations with the inclusion of Maqui flour (MF) at 5%, 7.5%, and 10%	Maqui improved the nutritional value, reporting a greater amount of nutrients, fiber, antioxidants, total polyphenolics, and total anthocyanins. In addition, cookies with Maqui flour were favored over the control ones, exhibiting a higher percentage of fiber. Compared with the control, anthocyanin content increased 3.5-fold and antioxidant levels increased approximately 10-fold.	[60]
Yogurt	Maqui berry powders (MBP)	4% and 8%	The perception that the consumer has about yogurt with Maqui powder (at 4% and 8%) could serve as potential prototypes for future market launches, given that they have an attractive color and present high levels of bioactive compounds, which makes the Maqui in a favorable option as a functional ingredient or food coloring.	
Meat patties	*Maqui leaf powders (Ma)*	500, 1000, 2000 ppm	Patties with Ma at 500, 1000, and 2000 ppm treatments demonstrated the lowest rates of lipid oxidation (42.05%, 40.29%, and 43.14%, respectively) compared to the synthetic antioxidant (52.23%). This is attributed to the high total polyphenol content (148.76 mg GAE/g), predominantly characterized by significant amounts of hydroxybenzoic acids (82.5 mg GAE/g), flavonoids (7.1 mg QE/g), and hydroxycinnamic acids (3.7 mg CAE/g). Organoleptic analysis revealed that the inclusion of Maqui leaf powders did not impact the overall acceptability of the new formulations.	[57]
Cookies	Maqui berry powders (MBP)	2.5%, 5%, 7.5%, and 10%	The incorporation of Maqui berry powders (MBP) increased the antioxidant capacity of the cookies. Concerning the color of the cookie surface, there was a significant decrease in L* and b* values, while the a* value increased with the addition of MBP. The most favorable sensory attributes and acceptability were observed with 7.5% of MBP. The analysis suggests that cookies with desirable physical characteristics and enhanced antioxidant activities can be achieved by substituting a portion of wheat flour with MBP.	[61]
Pasta	Maqui berry powders (MBP)	Replacement of durum wheat semolina with Maqui berry powder at 0, 7.5, and 15 g 100 g^−1^	Substitution of durum semolina with increasing levels of MBP resulted in shorter cooking time, increased firmness, and stickiness. The enriched pasta received a favorable acceptance score, surpassing the acceptability threshold. Pasta 7.5 (4.86 g/100 g) and pasta 15 (8.34 g/100 g) qualify for the “source of fiber” or “high fiber” claim, respectively. Predicted glycemic index values categorized pasta 7.5 and pasta 15 as low glycemic index pastas, indicating favorable outcomes in terms of starch digestibility for these products. Furthermore, the inclusion of MBP elevated the antioxidant capacity and total phenolic compound content, with pasta 15 showing particularly noteworthy results.	[62]
Drinks	Maqui berry powders (MBP)	Freeze-dried Maqui berries were incorporated into lemon juice to achieve final concentrations of 2.5% (*w*/*v*) and 5% (*w*/*v*) of ground fruit in the beverage.	Beverages formulated with Maqui berries and lemon juice exhibited protective interactions among bioactive phytochemicals and demonstrated good stability over time concerning the analyzed parameters. The presence of anthocyanins in Maqui berries played a crucial role in preserving vitamin C in lemon juice in these mixtures. Similarly, Maqui protected hesperidin and, consequently, the flavonoids present in lemon. Additionally, the newly developed beverages, enriched with bioactive phytochemicals, displayed substantial in vitro antioxidant activity and maintained an appealing and well-preserved color throughout the study period, particularly when stored at 4 °C.	[63]
Oil	Maqui leaf extract	Avocado oil was fortified with methanolic extract of Maqui leaves (OM) and ethyl ether extract of Maqui leaves (OE). The fortified oils were created with Maqui leaves (OE) at a concentration of 800 ppm.	Enhancing the thermo-oxidative stability of pure avocado oil is achieved through fortification with Maqui leaf extracts using two solvents. The methanolic extract exhibits a superior protective effect. This suggests that utilizing by-products of native plants, like leaves, could serve as an alternative to incorporating non-natural compounds into oils.	[59]
Cake	Maqui berry powders (MBP)	0.5, 1, 1.5, and 2.5%	The volume of the sponge cake decreased significantly with the increase in the substitution level of freeze-dried Maqui berry powder. Luminosity decreased significantly with the increase in freeze-dried Maqui berry powder in the crust and crumb of sponge cake. Hardness and stickiness increased, while gumminess tended to decrease with increasing Maqui berry powder. The consumer acceptability score of 0.5 and 2.5% freeze-dried Maqui berry powder obtained better acceptability results.	[64]

## 7. Conclusions and Future Trends

In the realm of emerging trends, Maqui finds new applications in food products, emphasizing the preservation of its antioxidant properties and the utilization of dietary supplements in cases of nutritional deficiencies. The nutritional evaluation suggests that these components can serve as nutraceuticals, exploring antioxidant-based molecules with diverse bioactive effects on human health, including antioxidant antihypertensive, antidiabetic, and anti-inflammatory properties. This paves the way for innovative strategies in designing nutritional supplements and functional foods. Maqui, known for its economic viability and numerous advantages, is witnessing substantial demand in the national and global markets across various sectors, such as plant-based medicine, food supplements, health products, pharmaceuticals, and cosmetics.

Simultaneously, ongoing research aims to replace wheat flour, either partially or entirely, with Maqui to create gluten-free products with unique nutritional characteristics. The current trend in Maqui usage focuses on improving the nutritional aspects of fortified products and positioning them as excellent supplements for treating various diseases, contributing to the creation of nutritious and nutraceutical foods.

In conclusion, bioactive compounds from Maqui demonstrate potential applications in the food industry. However, there is a shortage of results concerning the isolation of bioactive compounds, underscoring the necessity for further exploration to uncover new natural bioactive agents from Maqui plants and gain a deeper understanding of their role in the food matrix. Investigating these molecules represents a foundational step toward a comprehensive understanding of their function in the food matrix, facilitating accurate technological, nutritional, and sensory development of functional foods.

Additionally, there is a need to delve into the stability of nutrients and bioactive compounds in functional foods. Despite numerous studies reporting on the functional properties of bioactive compounds, there is insufficient research on the digestibility and bioavailability of these compounds in both in vivo and in vitro systems. It is imperative to conduct more clinical trials to demonstrate the functional properties of bioactive compounds present in Maqui.

This comprehensive review aimed to spotlight the bioactive compounds present in Maqui, exploring recent approaches to functional applications and their impact on the functional characteristics of functional foods.

Incorporating Maqui into food formulations enhances nutritional value by augmenting macro- and micronutrient contents, with a notable increase in bioactive fibers, vitamins, and minerals as it has been demonstrated previously with other natural extracts [65,66,67,68]. It is important to note that the choice between using Maquis seeds or leaves versus the fruit influences the lipid content, fiber, and proteins, with seeds contributing to higher fiber values, a characteristic not shared by the fruit. Nevertheless, elevated concentrations of Maqui can induce alterations in the physical and sensory characteristics of supplemented products. Cake, cookies, and juices have been the focal points of studies, providing valuable insights for future exploration in the realm of bakery products due to their shared ingredients and preparation methods.

While cookies have been the primary focus in most studies, Maqui has also found application in meats, juices, yogurts, and other food products. The incorporation of Maqui into the food industry stands as a significant contribution to enhancing the nutritional profile of various products. This strategy holds promise for improving the nutritional status and overall health. Additionally, it appeals to individuals seeking healthier food options, emphasizing that a food product should encompass more than just taste; it should convey meaning and contribute to overall well-being.

Currently, it is marketed in different formats, ranging from natural fruit to powdered and freeze-dried Maqui; however, there is also evidence of its use in the food industry as a nutritional and functional additive, as well as a stabilizer agent and an enhancer of sensory and organoleptic characteristics. Maqui has become an excellent functional food with great potential to be used in the pharmaceutical and food industries.

## Figures and Tables

**Table 7:** Recommended maximum consumption of Maqui berry powder and its raw material, according to the “Power of Maquiberry (*Aristotelia chilensis*)” dossier of the Food Safety Commission of the European Union.

Food	Intake (g of Maqui Berry Powder/100 g of Product)
Cereals and cereal products	
Bakery products	20 g/100 g
Cereals	30 g/100 g
Bread and rolls	15 g/100 g
Fine bakery wares	
Dietary foods for weight control diets intended to replace total daily food intake or an individual meal (the whole or part of the total daily diet)	
Beverages	
Coffee, herbal tea, and fruit infusions; coffee substitutes	5 g/100 g
Fruit nectars and vegetable nectars and similar products	5 g/100 g
Flavored drinks	5 g/100 g
Fruit juices and vegetable juices	5 g/100 g
Non-alcoholic beverages	5 g/100 g
Alcoholic beverages	5 g/100 g
Aromatized wine-based drinks	2 g/100 g
Other alcoholic drinks, including mixtures of alcoholic drinks with non-alcoholic drinks and spirits with less than 15% of alcohol	2 g/100 g
Dairy products	
Dehydrated milk	10 g/100 g
Flavored fermented milk products including heat-treated products	5 g/100 g
Confectionery	
Chewing gum	3 g/100 g
Includes all cocoa and chocolate products, other confectionery products that may or may not contain cocoa, chewing gum, decorations, and icings	5 g/100 g
Other confectionery including breath-freshening micro-sweets	2 g/100 g
Ready-to-eat savories and snacks (desserts)	15 g/100 g
Edible ices	5 g/100 g
Extra jam and extra jelly	30 g/100 g
Jam, jellies, marmalades, and similar products	30 g/100 g

## Data Availability

The original contributions presented in the study are included in the article, further inquiries can be directed to the corresponding author.
